# Completely thrombosed middle cerebral artery aneurysm mimicking cavernous malformation: illustrative case report with operative video and review of the literature

**DOI:** 10.1007/s00701-026-06841-3

**Published:** 2026-03-24

**Authors:** Beatrice Zucca, Marissa Koscielski, Aaron Kakazu, Sanjit Shah, Mary Gaskill-Shipley, Matthew Smith, Charles Prestigiacomo, Jonathan Forbes

**Affiliations:** 1 Alma Mater Studiorum-University of Bologna, Bologna, Italy; 2 Department of Neurological Surgery, University of Cincinnati Medical Center, 3113 Bellevue Ave, Cincinnati, OH 45219 USA; 3 University of Cincinnati College of Medicine, Cincinnati, OH USA; 4 Department of Neuroradiology, University of Cincinnati Medical Center, Cincinnati, OH USA; 5 Department of Neurology, University of Cincinnati Medical Center, Cincinnati, OH USA

**Keywords:** Thrombosed aneurysm, Middle cerebral artery, Cavernous malformation, Radiographic features

## Abstract

Complete aneurysmal thrombosis is a rare occurrence in which a combination of thrombus and plaque contributes to the complete obliteration of the lumen of an aneurysm, rendering it occult on angiographic imaging. To date, there is a paucity of information in the literature describing the clinical/radiographic presentation and operative management of complete thrombosed aneurysms of the middle cerebral artery. A focused literature review was performed to identify previously reported cases of complete MCA aneurysm thrombosis. Five cases meeting these criteria were identified, of which four underwent open microsurgical management and one was treated endovascularly. We report a clinical case of a 35-year-old female who presented with severe exacerbation of baseline headaches. Radiographic features were suggestive of a ruptured cavernous malformation. In the operating room, a large and completely thrombosed aneurysm of the superior M2 was encountered. The aneurysm was treated with ultrasonic evacuation of the thrombus and plaque, followed by definitive clip reconstruction. Completely thrombosed aneurysms are rare entities that can masquerade cavernous malformations (CCM) and/or intra-axial neoplasms. Following a literature review, we highlight nuances of diagnosis and discuss pearls of surgical management along with the inclusion of a narrated, operative video.

**Supplementary Information:**

The online version contains supplementary material available at 10.1007/s00701-026-06841-3.

## Introduction

Middle cerebral artery (MCA) aneurysms represent approximately 20–25% of all intracranial unruptured aneurysms (IUA) [3, 4]. Complete middle cerebral aneurysmal thrombosis is a rare occurrence whereby a combination of thrombus and plaque contributes to the complete obliteration of the lumen of an aneurysm, rendering it occult on angiographic imaging. While *partial* spontaneous thrombosis of cerebral aneurysms can occur in a small number of ruptured aneurysms and 10–30% of intracranial giant aneurysms (IGA), complete thrombosis of an MCA aneurysm is an exceptionally rare occurrence [2, 9]. As cerebral angiography by definition is unremarkable, completely thrombosed aneurysms are commonly mistaken for cavernous malformations (CCMs) or intra‑axial neoplasms on initial imaging.^.^ To date, only five reports of complete MCA aneurysm thrombosis have been documented in the literature. We present the case of a complete thrombosis of a superior M2 aneurysm that required ultrasonic removal of plaque and clip reconstruction for definitive treatment.

## Case report

A 35-year-old woman presented to an outside emergency department with severe exacerbation of baseline headaches that started approximately 6 weeks before seeking medical consultation. Initial non-contrast CT was negative for subarachnoid hemorrhage but demonstrated a hypodense lesion adjacent to the right Sylvian fissure. CT angiography of the head was negative for evidence of aneurysm or vascular irregularity. The patient was ultimately discharged home with plans to obtain elective MRI imaging.

**Fig. 1:**
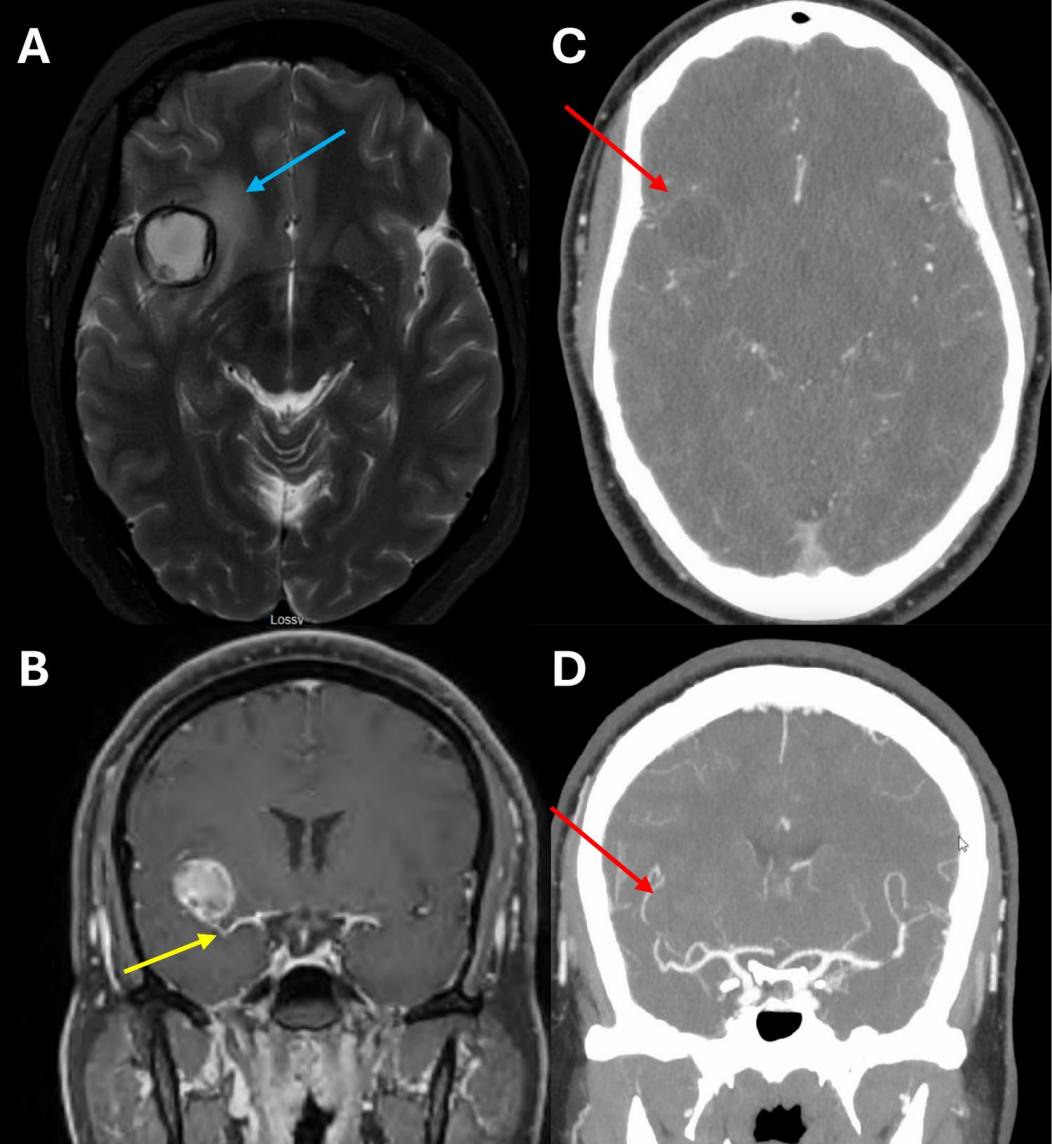
Pre-operative brain imaging demonstrates perilesional edema (blue arrow) on axial T2- weighted MRI of the brain (**A**), and post-contrast T1-weighted MRI (**B**) reveals involvement of the distal MCA bifurcation (yellow arrow). Axial (**C**) and coronal (**D**) CTA show no evidence of vascular irregularity (red arrows)

Outpatient MRI demonstrated a 2.3 cm rounded hemorrhagic lesion involving the right frontal opercular region and associated with minimal peripheral enhancement in post-contrast imaging. The hemorrhagic lesion involved the distal MCA bifurcation (Fig. 1). Pre-contrast T1 and T2-MRI weighted imaging defined a discrete hypointense rim and considerable peri-lesional edema. Following discussion in a multi-disciplinary setting, radiographic features were thought to be most consistent with a ruptured cavernous malformation. The possible etiology of complete thrombosis of MCA aneurysm was discussed, although it was deemed significantly less probable. Pre-operative diagnostic cerebral angiography (DSA) was not performed. Ultimately, a right frontotemporal craniotomy for definitive treatment was advocated. Intraoperatively, a large, completely thrombosed aneurysm of the superior M2 was encountered (operative video available in the Supplementary Material). The blood pressure was augmented in preparation for temporary clipping. Proximal and distal vascular control of the superior M2 was obtained with temporary clips. The aneurysm wall was opened sharply and treated with ultrasonic evacuation of thrombus and plaque followed by clip reconstruction. As motor-evoked potentials (MEPs) and somatosensory evoked potentials (SSEPs) remained stable until the end of the procedure, the temporary clips were only removed a single time to allow for reperfusion (with total temporary occlusion time of 17 minutes). After a very brief decline near the end of the phase of clip reconstruction, MEPs and SSEPs returned completely to normal after removal of temporary clips. Two long straight clips were used to disconnect the lumen of the aneurysm from the superior M2; these were buttressed by two fenestrated clips for circumferential vessel wall reconstruction. Subsequently, the dome of the aneurysm was sharply disconnected from the reconstructed aneurysm neck to de-vascularize the living aneurysm wall. Indocyanine Green (ICG) and doppler ultrasound confirmed appropriate preservation of the parent and daughter branches. At the completion of the procedure, all neuromonitoring signals were noted to be intact.

**Fig. 2:**
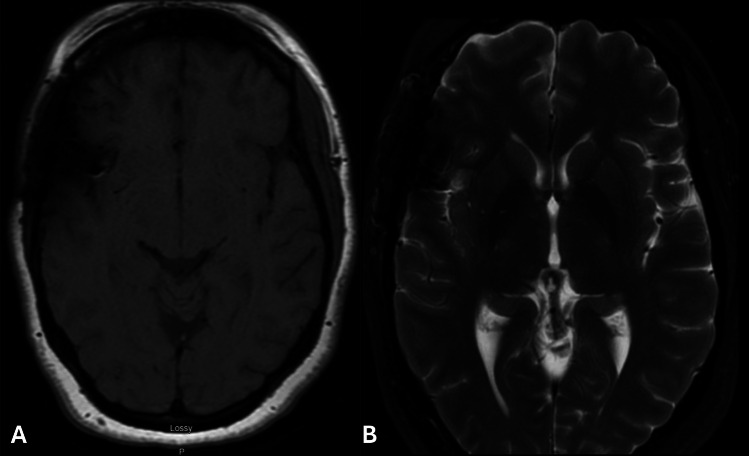
Post-operative brain imaging obtained two months after surgery demonstrates complete resection of the hyperintense lesion on axial T1-weighted MRI (**A**), while axial T2- weighted MRI (**B**) shows resolution of the perilesional edema

**Fig. 3:**
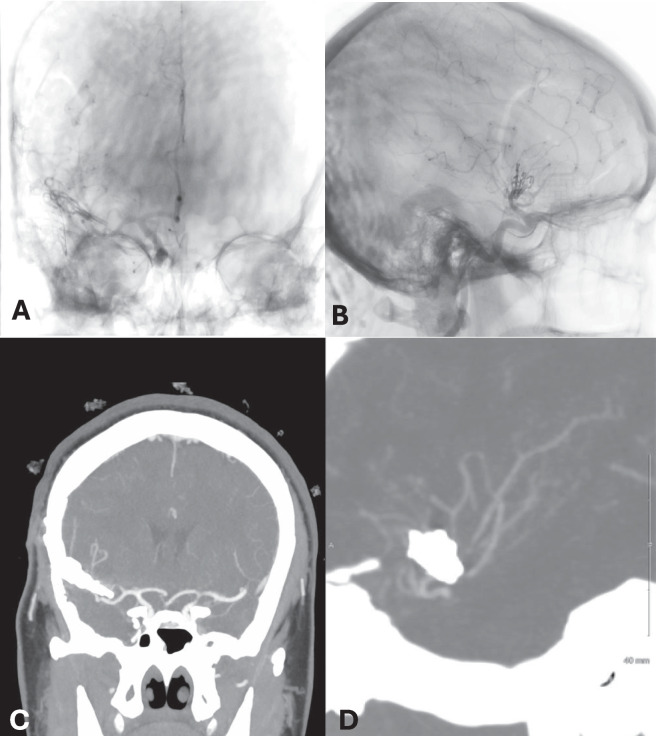
Post-operative DSA demonstrates complete preservation of flow of the reconstructed superior M2 division without luminal compromise or irregularity (**A-B**). 2-year post-operative coronal (**C**) and sagittal (**D**) CTA demonstrated preservation of the parent and daughter vasculature with no evidence of aneurysm recurrence

The patient had an uncomplicated postoperative course and was discharged home. At the three-month follow-up, she was neurologically intact and reported considerable improvement in baseline headaches. Post-clip MRI demonstrated resection of the T1 hyperintense focus with complete resolution of perianeurysmal T2 hyperintensity. (Fig. 2). Routine post-operative DSA performed in the elective setting following treatment demonstrated complete preservation of the parent and daughter vasculature (Fig. 3A-B). Post-operative CT angiogram obtained 2 years following treatment again demonstrated preservation of the parent and daughter vasculature with no evidence of aneurysm recurrence (Fig. 3C-D). This case highlights the diagnostic challenges and intraoperative findings posed by completely thrombosed aneurysms, which are angiographically occult and often misidentified before surgery. Full patient consent for photography and/or recording was obtained in the preoperative period.

## Discussion

Physiologically, aneurysm thrombosis is caused by low shear rates and the suppression of pulsatile flow, resulting in a procoagulant milieu. Complete aneurysmal thrombosis is a rare occurrence whereby a combination of thrombus and plaque contributes to the complete obliteration of the lumen of an aneurysm, rendering the lesion occult on angiographic imaging. The rarity of complete aneurysmal thrombosis, as well as the essential role angiographic imaging routinely plays in the diagnosis of aneurysmal pathology, makes accurate diagnosis of completely thrombosed aneurysms extremely challenging. Notably, in the existing reports detailing complete thrombosis of an MCA aneurysm, the majority of the lesions were misclassified as either cavernous malformations or intra-axial neoplasms prior to surgical intervention.

Given the inability to rely on angiographic imaging in the diagnosis of complete aneurysmal thrombosis, other radiographic features must be evaluated by the neurosurgeon. As a general recommendation, any time a lesion of unusual radiographic characteristics is found in a fissure or cistern typically occupied by large or medium sized vessels, the diagnosis of aneurysmal thrombosis should remain on the differential—even if angiographic imaging is negative. CT imaging of completely thrombosed aneurysms can reveal specific features such as peripheral ring enhancement, curvilinear mural calcifications, or heterogeneous high-density attenuation within the aneurysmal lumen. MRI features can sometimes include “onion skin” appearance due to layered signal intensities, heterogeneous signal within the lumen consistent with hemorrhage of different ages, marginal contrast enhancement involving the aneurysmal wall, and perianeurysmal edema on T2-weighted sequences [1, 7]. High-resolution vessel wall MR imaging, an emerging radiographic technique able to detect instability or inflammation within the vessel wall with higher sensitivity than traditional forms of MR imaging alone, was unfortunately not obtained in this case. However, the presence of marginal wall enhancement noted on traditional MR imaging was in hindsight perhaps indicative of an active biological process involving the arterial/aneurysm wall. While there is limited available data on the utility of high-resolution vessel wall MR imaging in the setting of completely thrombosed aneurysms, this technology offers hypothetical promise [7, 11]. As CT, MRI, and angiographic findings in thrombosed aneurysms are rarely pathognomonic, the surgeon must maintain a high index of suspicion for an accurate pre-operative diagnosis. Importantly, completely thrombosed aneurysms retain the potential to recanalize and evolve into partially thrombosed aneurysms, a configuration known to carry a significant risk of rupture. Prior studies have documented this phenomenon with systematic reviews reporting recanalization rates of up to 33% in non‑giant aneurysms, thereby underscoring the fact that thrombosis alone does not eliminate future instability [1].

The overwhelming majority of documented cases of aneurysm thrombosis in the literature involve partial or subtotal thrombosis. To date, only five cases of complete MCA spontaneous thrombosed aneurysms have been reported in the literature. Four of these cases were discussed in a review of the literature by Scerrati et al. that identified 94 cases of MCA aneurysms associated with some element of thrombosis [1, 5, 6, 8]. As an example of the rarity of completely thrombosed MCA aneurysms, 96% of the cases in the Scerrati report represented partially-thrombosed aneurysms [10].

Clinical and radiographic summaries of the five existing cases of completely thrombosed MCA aneurysms can be found in Table 1. Despite attainment of multiple imaging studies, three of the five cases were initially misdiagnosed, with one case classified as an intra-axial neoplasm and another case misdiagnosed as a cavernous angiomas. The results of open neurosurgical intervention for completely thrombosed aneurysms, performed in four of the five cases, demonstrate the complexity and morbidity associated with treatment of these lesions. In two of the four cases treated with open surgery, the identified aneurysm could not be clipped successfully and was treated with wrapping. In the remaining two cases treated with open microsurgery, one patient was treated with resection of the aneurysm and sacrifice of two daughter vessels. The other patient underwent partial debulking of the aneurysm with failure to definitively treat with clip reconstruction. In four of four cases of complete aneurysm thrombosis requiring open microsurgical intervention, aneurysm management was suboptimal with either incomplete treatment or daughter vessel sacrifice. In three of these four cases, suboptimal outcomes were at least partially attributed to the failure to recognize the aneurysm preoperatively. Review of these cases emphasizes the significance of accurate preoperative identification, as the primary therapeutic objective is complete aneurysm obliteration while preserving the flow of the parent and branching vessels.

**Table 1:** Completely thrombosed MCA aneurysms

Author, Year	Sex/Age	Symptoms	Imaging	Aneurysm Location	Initial Misdiagnosis	Treatment	Pre-Operative Diagnosis	Operative Outcome
Lenthall, 2001	F/36	Migraine, right-sided sensory weakness	DSA, MRI	M2 segment	N	Unable to clip partially thrombosed aneurysm; aneurysm wrapped	Partially thrombosed MCA aneurysm	Post-operative MRI demonstrated spontaneous progression to complete aneurysm thrombosis, patient subsequently observed
Cohen, 2003	M/25	Sudden headache and left arm weakness	CTA and DSA	MCA bifurcation	Y	Surgical intervention + endovascular embolization	Intracranial hemorrhage; Blister-like dilatation at MCA bifurcation of uncertain etiology	Unable to clip initially. Clipping aborted, wrapped with gauze. Aneurysm eventually recanalized requiring embolization
Kim, 2014	M/58	Headache and dizziness	CTA and DSA	Proximal MCA	N	Endovascular embolization after recanalization noted on DSA 2 months later	MCA aneurysm	Initial coil embolization aborted after aneurysm found to have completely thrombosed; subsequent coil embolization performed after aneurysm found to recanalize
Nguyen, 2015	F/43	Generalized tonic–clonic seizure	MRI, CTA and DSA	MCA (not specified)	Y	Surgical intervention	Intra-axial neoplasm	Resection of aneurysm with sacrifice of two distal vessels
Soler-Rico, 2023	M/40	Transient left hemiparesis and hypoesthesia	CTA and MRI	Distal MCA	Y	Surgical intervention	Cavernous angioma	Aneurysmal sac incision and partial debulking; unable to clip reconstruct; patient subsequently observed
Present case	F/35	Exacerbation of baseline headache	CTA and MRI	Distal M2 bifurcation	Y	Surgical intervention	Cavernous angioma	Aneurysm clipping with ultrasonic evacuation of thrombus

Consideration should be given to treatment of completely thrombosed MCA aneurysms, as they remain at risk for potential recanalization, instability, and delayed rupture. Given the small number of existing reports of completely thrombosed aneurysms, optimal treatment strategies are uncertain. Because these lesions are associated with complete angiographic obliteration of the lumen, endovascular intervention is not possible in the absence of delayed recanalization. Complete flow arrest with evacuation of plaque and thrombus and vessel wall reconstruction is thought to be an adequate management strategy that was successful in the index patient presented. Trapping of the aneurysm with bypass is an alternative strategy that may be associated with a lower risk of recurrence as compared to clip reconstruction alone. However, trapping and bypass requires extensive preparation and planning which is sometimes not possible due to many completely thrombosed aneurysms not being correctly diagnosed prior to operative exploration.

## Conclusions

Complete thrombosed aneurysms are challenging lesions that are commonly misdiagnosed prior to operative exploration. Surgeons must be conscious of the limitations of pre-operative imaging in identifying completely thrombosed aneurysms and be prepared to treat these lesions effectively when encountered.

## Supplementary Information

Below is the link to the electronic supplementary material.

Supplementary file1 Narrative video illustrating the surgical procedure (MP4 446137 KB)

## Data Availability

The data that support the findings of this study are available from the corresponding author, Beatrice Zucca, upon reasonable request.

## Abbreviations

CCM: Cerebral cavernous malformation
CT: Computed tomography
CTA: Computed tomography angiography
IGA: Intracranial giant aneurysm
MCA: Middle cerebral artery
MRI: Magnetic Resonance Imaging
DSA: Digital Subtraction Angiography

## References

[1] Cohen JE, Rajz G, Umansky F, Spektor S (2003) Thrombosis and recanalization of symptomatic nongiant saccular aneurysm. Neurol Res 25(8):857–859. 10.1179/016164103771953961 14669530 10.1179/016164103771953961

[2] Edner G, Forster DM, Steiner L, Bergvall U (1978) Spontaneous healing of intracranial aneurysms after subarachnoid hemorrhage. Case report. J Neurosurg 48(3):450–454. 10.3171/jns.1978.48.3.0450 632868 10.3171/jns.1978.48.3.0450

[3] Etminan N, Ruigrok YM, Hackenberg KAM, Vergouwen MDI, Krings T, Rinkel GJE (2025) Epidemiology, pathogenesis, and emerging concepts in unruptured intracranial aneurysms. Lancet Neurol 24(11):945–957. 10.1016/S1474-4422(25)00264-9 41109235 10.1016/S1474-4422(25)00264-9

[4] Fujimura S, Yanagisawa T, Kudo G et al (2025) Development and validation of a prediction model for intracranial aneurysm rupture risk. JAMA Netw Open 8(12):e2550772. 10.1001/jamanetworkopen.2025.50772 41632142 10.1001/jamanetworkopen.2025.50772 PMC12728650

[5] Kim HJ, Kim JH, Kim DR, Kang HI (2014) Thrombosis and recanalization of small saccular cerebral aneurysm : two case reports and a suggestion for possible mechanism. J Korean Neurosurg Soc 55(5):280–283. 10.3340/jkns.2014.55.5.280 25132936 10.3340/jkns.2014.55.5.280 PMC4130955

[6] Lenthall R, Rodesch G (2001) Complete thrombosis of a giant distal middle cerebral artery aneurysm. Interv Neuroradiol 7(3):263–267. 10.1177/159101990100700314 20663358 10.1177/159101990100700314 PMC3621066

[7] Martin AJ, Hetts SW, Dillon WP et al (2011) MR imaging of partially thrombosed cerebral aneurysms: characteristics and evolution. AJNR Am J Neuroradiol 32(2):346–351. 10.3174/ajnr.A2298 21087941 10.3174/ajnr.A2298 PMC3542832

[8] Nguyen HS, Doan N, Eckardt G et al (2015) A completely thrombosed, nongiant middle cerebral artery aneurysm mimicking an intra-axial neoplasm. Surg Neurol Int 6:146. 10.4103/2152-7806.164696 26425396 10.4103/2152-7806.164696 PMC4571614

[9] dos Santos MLT, Spotti AR, dos Santos RMT et al (2013) Giant intracranial aneurysms: morphology and clinical presentation. Neurosurg Rev 36(1):117–122. 10.1007/s10143-012-0407-0 22791075 10.1007/s10143-012-0407-0

[10] Soler-Rico M, Finet P (2023) Thrombosed MCA aneurysm mimicking an insular cavernous angioma: a case report and literature review. SN Compr Clin Med 5(1):279. 10.1007/s42399-023-01615-9

[11] Teng MMH, Nasir Qadri SM, Luo CB, Lirng JF, Chen SS, Chang CY (2003) MR imaging of giant intracranial aneurysm. J Clin Neurosci 10(4):460–464. 10.1016/S0967-5868(03)00092-4 12852886 10.1016/s0967-5868(03)00092-4

